# Immunogenic Cell Stress and Death Sensitize Tumors to Immunotherapy

**DOI:** 10.3390/cells12242843

**Published:** 2023-12-15

**Authors:** Oliver Kepp, Guido Kroemer

**Affiliations:** 1 Centre de Recherche des Cordeliers, Equipe Labellisée par la Ligue Contre le Cancer, Université de Paris Cité, Sorbonne Université, Inserm U1138, Institut Universitaire de France, 75006 Paris, France; 2 Metabolomics and Cell Biology Platforms, Gustave Roussy Cancer Center, 94800 Villejuif, France; 3 Department of Biology, Institut du Cancer Paris Cancer Research and Personalized Medicine (CARPEM), Hôpital Européen Georges Pompidou, Assistance Publique-Hôpitaux de Paris (AP-HP), 75015 Paris, France

The efficacy of chemotherapy with cytotoxicants and that of targeted therapies with more sophisticated agents is limited due to the plasticity of malignant cells, which leads to the inevitable development of resistance. Immune checkpoint inhibitors (ICIs) that target inhibitory cytotoxic T lymphocyte associated protein 4 (CTLA4) or T cell exhaustion signals such as programmed cell death-1 (PDCD1, better known as PD-1) and programmed cell death-1 ligand 1 (CD-274, better known as PD-L1) occupy a central stage in the first-line adjuvant and neoadjuvant treatment of advanced neoplasms [1,2]. ICIs are now broadly used through the oncological spectrum.

Nevertheless, certain cytotoxic agents have the ability to induce durable disease control exceeding the clinical treatment phase in many cancer patients. Retrospectively, it appears that those agents that were empirically selected by clinicians for decades, due to their effectiveness, are particularly capable of triggering specific stress and death pathways in cancer cells, rendering them recognizable to the immune system [3,4]. In preclinical mouse models, treatment of tumors with drugs inducing immunogenic cell death (ICD) is only efficient if functional dendritic cells (DCs) and T lymphocytes are available [5,6,7]. In patients, the induction of ICD correlates with the recruitment of antigen-presenting cells (APCs) and cytotoxic T lymphocytes (CTLs) into the tumor bed, and tumor infiltration by DCs and CTLs is indeed a biomarker of favorable prognosis [8,9,10]. ICD inducers include conventional chemotherapeutic agents such as anthracyclines, oxaliplatin, and taxanes, as well as more disease-specific targeted agents from the group of tyrosine kinase inhibitors (TKIs) [11,12]. Moreover, ionizing irradiation [13,14], photodynamic therapy (PDT) [15,16], and oncolytic viruses [17,18] have been shown to induce ICD. In preclinical experimentation and in clinical routine, it appears that combinations of ICD inducers with immune checkpoint inhibitors (ICIs) are particularly efficient in mediating their therapeutic potential [19,20].

ICD facilitates the onset of anticancer immune responses via an increase in both the antigenicity as well as the adjuvanticity of malignant cells. Mechanistically, this involves the onset of coordinated premortem stress responses that can affect the antigenic makeup of cancer cells via genetic or epigenetic alterations of the transcriptome. Moreover, ICD-related cellular stress facilitates the emission of normally confined ‘danger associated molecular patterns’ (DAMPs) that act on the pattern recognition receptors (PRRs) expressed on professional antigen DCs [21,22,23,24]. Thus, immature DCs which express the ATP receptor P2Y2 are chemotactically attracted to the tumor bed by ATP secreted from cancer cells undergoing ICD [15,25]. The final approximation of DCs towards dying cancer cells is facilitated by tumor-emitted annexin A1 (ANXA1), which acts on the formyl peptide receptor-1 (FPR1) present on DCs [24]. Antigen transfer from tumor cells to DCs is mediated by an ‘eat-me’ signal, namely surface-exposed calreticulin, that appears on the plasma membrane of malignant cells and then acts on its receptor CD91, which is present on DCs [13]. The maturation of DCs is ignited via the ligation of Toll-like receptor 4 (TLR4) by the high mobility group box 1 (HMGB1) emanating from the nuclear compartment of dying cancer cells. DC maturation further drives the production of type-1 interferons, which in turn amplifies the synthesis of the C-X-C motif chemokine ligand 10 (CXCL10), thus stimulating T cell priming. In sum, the induction of ICD in malignant cells leads to a coordinated alteration of the cell surface and the local secretome, thereby facilitating the attraction, differentiation, and maturation of DCs so that they present tumor-associated antigens to T cells and, hence, initiate adaptive anticancer immunity [22,23,24]. Of note is that malignant cells and pathogenic viruses can subvert ICD-associated DAMP emission, thus blunting the immune response. Moreover, inherited or acquired defects in the perception of ICD by the host immune system can undermine immunosurveillance and provoke failure of cancer treatments.

The concept of ICD has transcended the realm of preclinical experimentation and is now used for drug discovery (to identify novel ICD inducers) for the design of clinical trials (to identify suitable drug combinations, particularly with ICIs). The present Special Issue, “Immunogenic Cell Stress and Death”, discusses different strategies for inducing anticancer immunity by facilitating the molecular crosstalk between cancer cells, DCs, and T lymphocytes. The role of ICD is reviewed in the context of colorectal, gastric, pancreatic, and hepatocellular cancer as well as multiple myeloma. Moreover, it is revealed that combination treatment with cetuximab plus cisplatin for the treatment of head and neck cancer induced traits of ICD. Interestingly, high doses of the non-immunogenic cell death inducer cisplatin blunted antitumor immunity in this context. Additional aspects of immunogenic cell stress and death cover the role of ICD-associated chemokines and chemokine receptors in the activation of CD8 T-cells and clinical applications thereof. Furthermore, light is shed on the role of tumor-associated macrophages (TAMs) in the response to dying cancer cells. Finally, three-dimensional organ-on-chip technology is introduced for modeling the tumor microenvironment (TME). At the mechanistic level, evidence is presented that targeting the unfolded protein response (UPR) can increase the efficacy of anticancer therapy.

In this Special Issue, ICD induction is discussed in different disease-relevant therapeutic approaches, novel immune signals are described, and the roles of specific immune cell subtypes are elucidated. Advanced in vitro systems help us understand the apical targets of ICD, the organellar genesis of ICD signals, and the complex cellular interplay in the tumor microenvironment. Altogether, an ever more sophisticated pipeline of preclinical ICD-relevant exploration will prepare the basis for the clinical implementation of ICD-based anticancer therapies.
